# STEM Tools for Semiconductor Characterization: Beyond High-Resolution Imaging

**DOI:** 10.3390/nano12030337

**Published:** 2022-01-21

**Authors:** María de la Mata, Sergio I. Molina

**Affiliations:** Departamento de Ciencia de los Materiales e Ingeniería Metalúrgica y Química Inorganica, IMEYMAT, Universidad de Cádiz, 11510 Puerto Real, Spain; sergio.molina@uca.es

**Keywords:** STEM atomic-resolution, spectroscopy, VEELS, 4D-STEM, electric field mapping, in situ, opto-electronic properties, semiconductor nanostructures

## Abstract

The smart engineering of novel semiconductor devices relies on the development of optimized functional materials suitable for the design of improved systems with advanced capabilities aside from better efficiencies. Thereby, the characterization of these materials at the highest level attainable is crucial for leading a proper understanding of their working principle. Due to the striking effect of atomic features on the behavior of semiconductor quantum- and nanostructures, scanning transmission electron microscopy (STEM) tools have been broadly employed for their characterization. Indeed, STEM provides a manifold characterization tool achieving insights on, not only the atomic structure and chemical composition of the analyzed materials, but also probing internal electric fields, plasmonic oscillations, light emission, band gap determination, electric field measurements, and many other properties. The emergence of new detectors and novel instrumental designs allowing the simultaneous collection of several signals render the perfect playground for the development of highly customized experiments specifically designed for the required analyses. This paper presents some of the most useful STEM techniques and several strategies and methodologies applied to address the specific analysis on semiconductors. STEM imaging, spectroscopies, 4D-STEM (in particular DPC), and in situ STEM are summarized, showing their potential use for the characterization of semiconductor nanostructured materials through recent reported studies.

## 1. Why Choose STEM

Advances in the development of novel and improved functional materials require deep characterization analyses to fully understand and exploit their physical properties. Within this context, transmission electron microscopy (TEM) is a powerful tool providing a broad variety of techniques, which allow in-depth analyses of the materials’ micro-/nano-/atomic structure and composition, as well as addressing some related physical properties.

TEM techniques may be first catalogued as those performed illuminating the region of interest using a parallel electron beam (TEM), and those employing an electron beam probe scanning the area of interest (scanning TEM, STEM). Conventional and high-resolution (HR-) TEM imaging relies on diffraction and phase-contrast imaging of the transmitted electrons, providing a comprehensive picture of the microstructure of the materials. STEM-related techniques take advantage of different emerging signals from the electron–matter interaction while scanning the electron probe over the sample, including chemical or opto-electronic analyses, among others.

The emergence of novel electron sources, with increased brightness and stability, along with the appearance of aberration correctors and electron monochromators, have driven the development of advanced methodologies, particularly in STEM mode. The lat-est advances in terms of faster and better detectors, enabling detection down to a single direct electron with high speed read-outs [2], and the implementation of computational schemes for data processing, widen the attain-able information accessible by STEM-related techniques. The of upcoming new detector designs, such as segmented and pixelated detectors whose components (segments or pixels) work independently collecting one dataset at every scanned position, has pushed the development of 4D-STEM. As we will see, these data collections allow for virtual imaging, ptychography, and electromagnetic field imaging, among others (see Section 2.2).

Another primary distinction among imaging techniques distinguishes between bright field (BF) and dark field (DF) techniques, depending on whether the collected electrons are on-axis or off-axis, respectively, referred to the electron microscope optical axis. Interestingly, the image formation in HRTEM and BF-STEM relies on the same electron pathway with opposite trajectories, which is known as the principle of reciprocity [1]. Nowadays, most electron microscopes can be easily operated under both modes reaching atomic resolution; however, the ultimate state-of-the-art usually involves dedicated instruments with enhanced stability. Other than that, further concepts to take into account are elastic and inelastic electron scattering, which refers to electrons keeping their energy when crossing the sample and those losing energy, respectively; and the relative coherence of the collect-ed electrons, leading to coherent or incoherent signals (i.e., in-phase and out-of-phase sig-nals, respectively). Accounting for the abovementioned factors, STEM offers a variety of imaging techniques, highly useful for structural analysis, including microstructure, crys-tal distortions/defects/lattice strain, etc. (Section 2.1).

Importantly, different electron–matter related signals can be monitored while work-ing in STEM. Therefore, different spectroscopic techniques can be implemented to address both, composition and opto-electronic properties. On one hand, measuring the energy lost by inelastically scattered electrons (Electron Energy Loss Spectroscopy, EELS) is an out standing technique with chemical capabilities, attaining information on bond-ing/coordination/oxidation states while it is also suitable to address opto-electronic properties and even vibrational modes (Section 3.1). On the other hand, the composition and lumi-nescent performance of the materials can be addressed by energy dispersive X-ray spec-troscopy, EDX, and cathodoluminiscence, CL, respectively (Section 3.2). Current EDX detectors with large solid angles reach enhanced efficiencies and improved quantification performance inside the TEM column, while STEM-CL is suitable with a detection system consisting of a parabolic mirror and a spectrophotometer, achieving spatial resolution down to the nanometer.

Moreover, either in situ sample holders, equipped with chips for electrical bias-ing/heating, etc., or environmental microscopes with sample chambers operating at high-er pressures than that of the TEM column, impose additional milestones for the study of nanoscale dynamic processes (see Section 4).

It is worth mentioning the relevant role of computational schemes to handle and un-derstand the obtained results. Software improvements and updates oriented to enhance acquisition and analysis skills are continuously growing. Particularly, machine learning approaches, due to their prediction ability, are a powerful tool within this context, with applications from structural analysis to data denoising, among many others (see Section 5).

Thereby, the unique capabilities of STEM along with the many operational modes render an ideal tool to face countless correlative studies, thanks to the struc-ture–properties relationships attainable. In the case of semiconductor materials, there are many studies focusing on STEM characterizations to cover their structural properties, in-cluding structural defects, atomic ordering, polarity, and related phenomena such as growth mechanisms, strain relaxation, and quantum structures, as well as those ad-dressing the electronic and opto-electronic properties, as reviewed elsewhere [3] for the case of 1D nanostructures (nanowires). Further than that, related 4D-STEM techniques and specifically designed methodologies combining several techniques have earned popularity over the last years. Zamani et al. [3] devoted their review specifically to semiconductor nanowires, paying particular attention to structural studies focusing on, for instance, the determination of growth mechanisms or lattice strain. We have expanded the scope to semiconductor nanostructured materials, including 2D semiconductors, spreading the discussion over a combination of techniques, 4D-STEM, spectroscopies, and in situ measurements.

**Figure 1:**
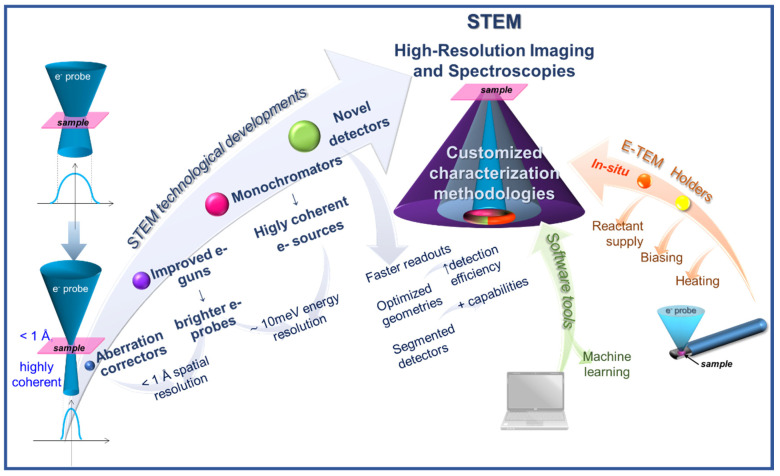
Recent developments contributing to the current powerfulness of STEM, a many-fold characterization tool with high spatial and spectral resolution capabilities.

The present review shows the potential of STEM techniques applied to semiconductor materials, ranging from atomic resolution imaging to electric field measurements, covering a variety of spectroscopies and in situ analyses to fully address the structure, composition, and functionality of the materials. The referred STEM techniques are briefly explained, and some of the last instrumental advancements (Figure 1) and derived novel or updated techniques are presented, as well as several procedures based on the combination of different techniques. The recent chosen publications exemplify the outstanding role of STEM methodologies applied to semiconductor materials, whilst paving the way for the development of specific strategies for further analyses.

## 2. STEM Imaging Techniques

### 2.1. Annular Detectors

**Figure 2:**
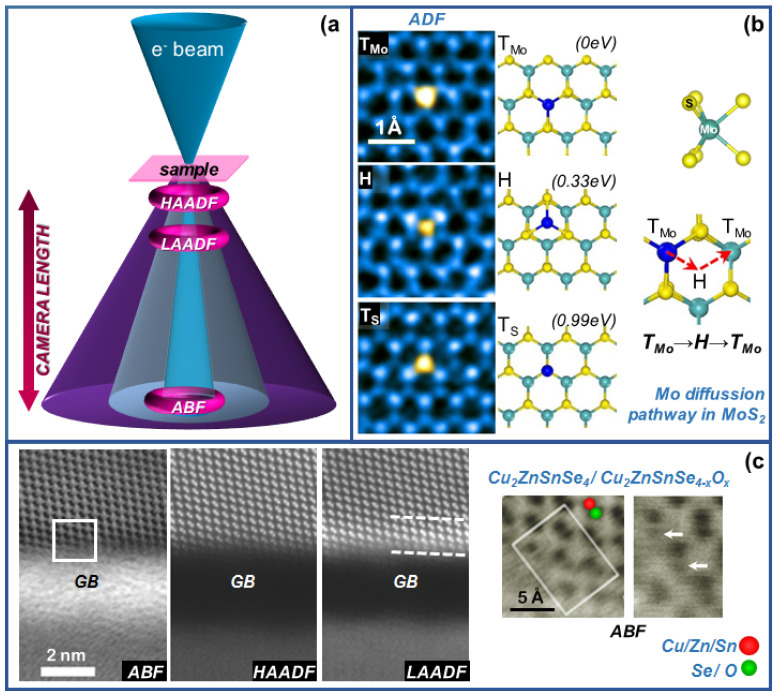
(**a**) Different techniques achievable at atomic resolution by means of annular detectors (HAADF, LAADF, ABF). (**b**) Atomic-resolution ADF images (left) and corresponding atomic models (middle) of different adsorption states of a Mo atom on top of Mo site (T_Mo_), at hexagon center or hollow site (H) and on top of S site (T_S_), from top to bottom. DFT calculated energy for each state is indicated between brackets (right). Most likely pathway for Mo diffusion in MoS_2_, T_Mo_ → H → T_Mo_. S and Mo are represented by yellow and cyan spheres, respectively. Adapted with permission from ref [6]. Copyright 2017, American Chemical Society. (https://pubs.acs.org/doi/abs/10.1021/acs.nanolett.6b05342, 9 December 2021). (**c**) Polycrystalline Cu_2_ZnSnSe_4_ thin films showing an amorphous intergranular oxide phase (grain boundary, GB). O substitutes some Se sites near the grain boundary, leading to the observed strain contrast (LAADF image) arising from compositional differences (O content). Adapted with permission from ref [7].

The imaging capabilities of STEM techniques have been widely exploited over recent years in material science, providing valuable insights into subtle structural features, which influence the system properties. As already mentioned, the emergence of electron beam probe correctors and brighter electron sources enable atomic-resolution imaging in STEM mode under a variety of techniques. Probe correctors compensate for the aberrations of the electromagnetic lenses driving the electrons from the source towards the sample, providing sub-angstrom electron probes to scan the area of interest, which results in atomic resolution imaging [4]. These techniques rely on the use annular detectors placed at suitable collection angles, attainable by setting the proper camera length (Figure 2a). Early annular detectors were designed to allow the simultaneous acquisition of images and spectra while performing electron energy-loss spectroscopy (EELS): The annular detector gathers elastic scattered electrons to provide an annular dark field (ADF) image, while the spectrometer collects the inelastic scattered electrons as a function of the energy lost (see Section 3.1) [5]. Importantly, different physically meaningful signals may be acquired depending on the collection angle of the annular detector (i.e., elastically or inelastically scattered coherent/incoherent electrons), rendering information on the sample structure, composition, microstrain, etc.

Modern electron microscopes are usually equipped with at least two annular detectors with adjustable detection ranges, leading to the simultaneous performance of different techniques. The most commonly employed STEM imaging technique is high-angle annular dark field, HAADF, also referred to as Z-contrast imaging since the image contrast scales with the atomic number, Z, of the sample constituents as follows:

(1) $$I\propto Z^{\alpha }$$

Although α values range from 1.2 to 1.8 as a function of the actual collection angle [8], for many practical purposes, α = 2 can be assumed. Therefore, the observed contrast intensity is directly related to the chemical composition, allowing straightforward phase identification and containing quantitative information. The technique relies on the collection of incoherent elastically scattered electrons (Figure 2a) at angles within β = 70–200 mrad (see Table 1). Performing the technique at an atomic resolution enables the detection of individual atoms [9], and the chemical quantification of individual atomic columns [10], remarkably interesting for the study of ternary/quaternary semiconductor compounds. Indeed, different methods have been developed for quantitative annular dark-field, useful for many applications [11]. For instance, specific methodologies have been developed to quantify the composition of III-V alloys [12], convenient for the evaluation of atomic composition modulations driving the formation of quantum dots. [13,14] Accurate quantitative analyses are possible by optimizing convergence and collection angles depending on the sample thickness, while keeping lowest image sampling and electron doses [15].

Notably, there are many methodologies based on the analysis of atomic resolution images, which cannot be covered in detail here. For instance, atomic resolution images contain information on the lattice strain and relaxation mechanisms known to impact the performance of semiconductors, which can be analyzed by different approaches, as geometric phase analysis (GPA) [16] or peak pair analysis (PPA) [17]. It is also important to note that structural and morphological information on the third spatial direction is accessible by tomographic reconstructions, based on processing 2D images of the same area of interest under different projection tilts, highly useful for the study of complex morphologies and heterostructured systems [18,19].

The poor imaging ability of HAADF to visualize light atoms due to their weak scattering power, especially when combined with heavier elements, drove the popularity of annular bright field (ABF) few years ago, proving its capability of imaging even the lightest element [20]. In this case, the required collection angles are β = 11–22 mrad to detect inelastically scattered electrons (Table 1) lying within the bright field region (Figure 2a). In order to identify unequivocally the atomic columns, ABF and HAADF signals can be simultaneously acquired, rendering complementary information. It should be noted that BF imaging including unscattered electrons by covering large collection angles (β ≈ 0–22 mrad), known as large-collection-angle bright field, is also suitable for chemical identification at atomic scale [21]. Alternatively, BF imaging can be performed selecting part of the whole BF region, leading to low-angle bright-field (LABF), middle-angle BF (MABF), and high-angle BF (HABF). While LABF-STEM imaging results from the interference of direct and diffracted beams entering the detector, showing contrast reversals similarly to HRTEM imaging, HABF presents inverted image contrast compared to HAADF-STEM. Placing the BF detector between HABF and LABF allows MABF imaging, where light and heavy atoms appear as bright and dark spots, respectively [22].

There are other useful angular collection ranges other than HAADF and ABF, such as low-angle annular dark-field, LAADF. Under LAADF experimental conditions, involving DF of partially coherent scattered electrons (collection ranges around 35 mrad, see Table 1), the resulting image provides defect contrast likely as consequence of de-channeling effects [23]. As in the case of ABF, LAADF signal can be acquired at the same time than HAADF, over the same area, with it even being possible combining the three of them [7].

The simultaneous acquisition of atomic-resolution images at different collection angles entails huge advantages for the design of specific methodologies. For instance, III-V semiconductors have been widely studied over recent years under HAADF-ABF atomic-resolution conditions to address the relative position of the atomic species within the crystal lattice of binary compound nanostructures [24]. The extracted data is highly valuable to explain the growth mechanism of semiconductor nanostructures, polarization effects, optical properties or piezo-electric behavior [25], to list some examples. Detailed structural and chemical analysis can be conducted at heterointerfaces, including in-depth studies of structural defects [26]. Facing more complex semiconductor alloy compositions, where every atomic column may be composed by more than one atomic specie, i.e., quaternary III-V alloys, as dilute nitrides, requires from other analytical strategies. A convenient solution may be found by combining LAADF and HAADF, as the static atomic displacements induced by atomic substitutions in alloys (i.e., N atoms in dilute nitrides) are trackable by LAADF, while HAADF provides Z-contrast complementary information [27]. Importantly, when dealing with quaternary III-V alloys, the crosstalk effect between neighboring atomic columns must be considered to reach reliable HAADF quantitative information [28]. Alternatively, more than two imaging modes can be combined together, such as ABF, HAADF and LAADF, rendering suitable tools to address substitutional lattice impurities, such as oxygen near surfaces or defects [7]. For example, Figure 2c summarizes the results extracted from atomic resolution analysis of polycrystalline Cu_2_ZnSnSe_4_ thin films, which contain amorphous intergranular oxide phases (grain boundaries, GB). The characterization under ABF, HAADF and LAADF reveals the formation of Cu_2_ZnSnSe_4−x_O_x_ owing to O placed at Se sites near the grain boundary, leading to the observed strain-contrast arising from compositional differences (O content).

Excitingly, atomic-resolution STEM imaging, supported by ab initio calculations, can be applied to the study of dynamic processes such as atomic diffusion. Tracking the migration of atomic species or vacancies by means of sequential imaging (time-lapsed) allows for unrevealing the atomic pathways of diffusion phenomena, as in the case of a single Mo atom in MoS_2_ (Figure 2b) [6]. Three different adsorption states are distinguished for the moving Mo atom, on top of a Mo site (T_Mo_), at the hexagon center of the MoS_2_ structure and on top of a S site (T_S_), classified as ground state (T_Mo_) and metastable states (H, T_S_) according to their relative DFT-calculated energies. The most likely pathway for Mo diffusion within MoS_2_ is through the metastable H state.

These few examples illustrate how atomic resolution STEM imaging provides a highly tunable tool, suitable to establish specific strategies to assess the quantitative/qualitative characterization of semiconductor nanostructured materials at atomic level.

### 2.2. Segmented Detectors: 4D-STEM

The development of newer detectors based on recent technologies enables faster signal readouts over high dynamic ranges, rendering increased sensitivity while minimizing the electron dose irradiation. Novel electron detectors can be designed based on different geometries, such as quadrant-segmented annular detectors and pixelated detectors [29], suitable for 4D-STEM data set collection [30]. While conventional STEM detectors record one single signal for every probe position, divided detectors collect split signals corresponding to the differentiated detector segments for each scanned electron probe position.

4D-STEM measurements allow imaging electromagnetic fields, providing a valuable tool for countless applications, from multiferroics [31] to skyrmions, including p-n junctions [32], quantum wells [33], magnetic domains [34] or giant polarization [35], among others. The simultaneous acquisition of electron diffraction (ED) patterns for every electron probe position along with ADF images allows many different post-data reconstructions, including different STEM annular imaging modes (BF, ABF, LAADF, HAADF), electric field maps, ptychography [36,37], differential phase contrast (DPC), or integrated differential phase contrast (iDPC) [38]. The attainable results reach atomic resolution when using sub-angstrom electron probes for the analyses [39,40,41], providing information even inside single atoms [42], with potential applications, for instance, in atomic defect detection (sub-lattice sites, vacancies and dopants) or for the study of local electrostatic properties, achieved by mapping the local projected electric field.

**Figure 3:**
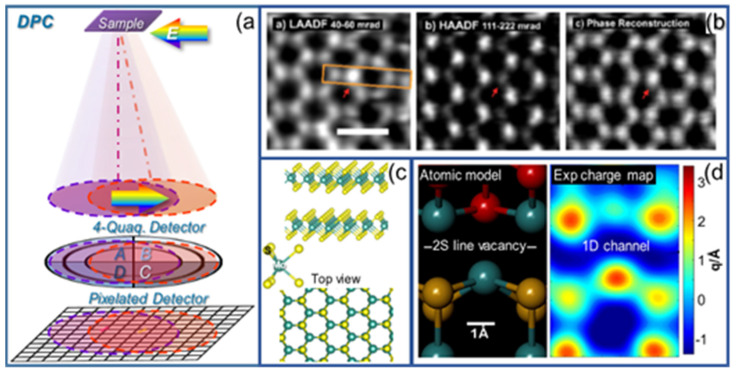
(**a**) Principle of DPC imaging (top). Electron pathway in presence of electric fields (deflection), recorded by 4-quadrant (middle) and pixelated (bottom) detectors. (**b**) 4D reconstructed LAADF, HAADF, and phase reconstruction images of a MoS_2_ monolayer showing an extra atom at 2S site. Adapted with permission from Y. Wen et al., 2019, Nano Lett. **19**, 6482. Copyright 2019 American Chemical Society (ref. [38]). (**c**) Atomic structure (3D model) of MoS2, along with its 2D projected top view (along c-axis). (**d**) 1D MoS2 line defect (2S line vacancy) atomic model (left) and experimental charge map (right) showing a 1D electron-rich channel (q/Å <0, dark blue) at the Mo–Mo bonding area where the line defect is located. Adapted with permission from S. Fang et al., 2019, Nano Lett. **19**, 6482. Copyright 2019 American Chemical Society (ref. [44]).

Among the variety of 4D-STEM techniques, ptychography provides an outstanding dose-efficiency approach suitable for image reconstruction. The technique retrieves phase information from the desired frequency range extracted from diffractogram datasets [43]. The ability to recover atomic resolution images over large fields of view implies an additional advantage of the technique. Furthermore, ptychography reconstructions combined with angular dependent ADF-STEM reconstructions can image simultaneously low and high atomic number elements, which is useful, for instance, for dividing atomic columns with similar total atomic number into their relative combinations, or for the identification of surface adatoms [38]. Advantageously, the virtual imaging capabilities, covering the whole angular range, allow for exploring the angular dependence of ADF image contrast, as illustrates Figure 3b, evidencing plain differences in the contrast of an interstitial atom at 2S site in a MoS_2_ monolayer, imagined under LAADF, HAADF and phase imaging conditions [38].

The assessment of electromagnetic properties by means of 4D-STEM relies on the electron deflection induced by electromagnetic fields, addressable by differential phase contrast imaging, DPC. The technique measures differences in the signals arising from opposite segments, due to the movement of the center of mass (COM) of the convergent beam electron diffraction (CBED) pattern, being linearly proportional to the (projected) electric field. Therefore, the recorded DPC signal corresponds to the COM movement, containing phase information. Figure 3a illustrates the electron deflection (i.e., displacement of the diffracted electron beam, which impinges on the detector, making it off-centered) induced by the presence of an electric field. Hence, decomposing the detected signal into orthogonal components using segmented/pixelated detectors provides insight into the electromagnetic field, allowing the study of related properties. Note that by considering diametrically opposed segments, the electric and magnetic fields can be separately analyzed [33,34]. Interestingly, DPC measurements with sub-angstrom resolution are also attainable using a complementary metal–oxide–semiconductor (CMOS) camera instead of a pixelated detector, as shown elsewhere [41].

The 2D integration (integrated DPC, iDPC) of the two components of the DPC image, leads to phase retrieval [45], and it captures the shift of the electron wave, φ(r), directly related to the electrostatic potential, Φ(r):

(2) $$\varphi \left(\vec{r}\right)=\sigma \;\Phi \left(\vec{r}\right)$$

where $\sigma =2\pi \mathrm{me}\lambda /h^{2}$ (m: electron mass, e: electron charge, λ: electron wavelength). In other words, DPC shows the electric field vector, whose integration (iDPC) results in the scalar electrostatic potential, Φ(r). In addition, phase measurements of the transmitted electrons in STEM lead to address the projected electric field, E(r) and the projected charge density, ρ(r), linked through Poisson’s equation and Gauss’ law to the phase gradient and its Laplacian, respectively [45]:

(3) $$\nabla^{2}\Phi \left(\vec{r}\right)=-\vec{\nabla }\cdot \vec{E}\left(\vec{r}\right)=-\frac{1}{\varepsilon_{0}}\;\rho \left(\vec{r}\right)$$

where $\varepsilon_{0}$ is the vacuum permittivity.

The key role of electromagnetic fields on the performance of many semiconductor systems turns this methodology into a convenient tool to test their behavior. For instance, DPC can be used for the identification and analysis of p–n junctions by imaging the built-in electric field at heterointerfaces [32]. The methodology has become increasingly popular due to its high-resolution capabilities, along with its large field of view. Atomic resolution analysis can be successfully implemented to common III-V semiconductors, addressing atomic electric fields, charge, and electron densities [46,47]. The measurable atomic electric fields render high contrast atomic resolution, which is appealing for light element imaging. It must be noted that flat thin specimens are required for the analysis, as they avoid thickness-variation induced artefacts. Therefore, 2-D layered materials, such as transition metal dichalcogenides (MX_2_, X = S, Se, Te), TMDs, offer an ideal playground for implementing DPC-STEM. For example, sub-angstrom resolution studies have proven electrostatic field fluctuations related to the location of crystal defects in TMD monolayers, where in-line vacancies lead to electron-rich areas of 1D conduction channels (see Figure 3d) [44].

## 3. Spectroscopy in STEM

**Figure 4:**
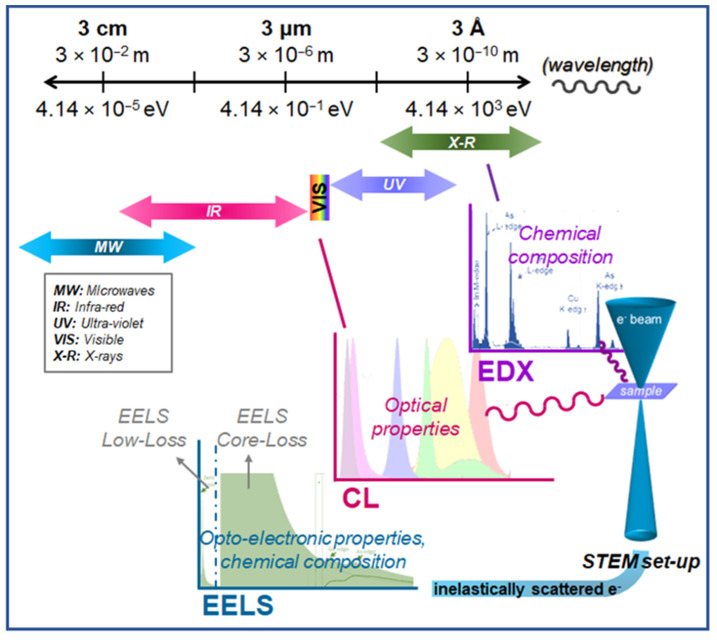
Spectral range covering wavelengths from 3cm to 3Å, along with STEM-related spectroscopies suitable for the characterization of semiconductor nanostructures, such as Energy Dispersive X-ray (EDX), Cathodoluminiscence (CL), and Electron Energy-Loss Spectroscopy (EELS). Each technique is displayed according to the wavelength (top scale) of the monitored signal (i.e., X-rays, photons, and inelastically scattered electrons for EDX, CL, and EELS, respectively).

In addition to the broad variety of imaging techniques available under STEM conditions, spectral analyses are also feasible. Actually, STEM provides a powerful analysis tool due to the many different chemical and opto-electronic related signals emerging while scanning the electron probe over the sample. A highly energetic electron beam impinging on a material produces different signals, such as photons, excitons, phonons, X-rays, secondary electrons, etc., as well as diffracted and scattered electrons, which are measurable by fitting the appropriate detectors in the experimental setup. Figure 4 summarizes the main spectroscopies attainable working in STEM, as a function of the wavelength, ranging from X-rays (lower wavelengths) to infrared radiation, including the UV-VIS spectral range.

In the following, we present different STEM spectroscopies, namely electron energy-loss spectroscopy, EELS, energy dispersive X-ray, EDX, and cathodoluminiscence, CL, showing the latest breakthroughs regarding semiconductor materials.

### 3.1. Electron Energy-Loss Spectroscopy (EELS)

Electron energy-loss spectroscopy measures the kinetic energy of transmitted electrons after crossing the material sample [48], providing information on the energy distribution of the electrons due to inelastic scattering. Inelastic scattering accounts for the energy exchange between impinging upon electrons and those belonging to the atomic sample constituents. Energy filter systems equipped with electromagnetic prisms enable the spreading of the unscattered and inelastically scattered electrons according to their lost energy. The most common filter designs include four electromagnets arranged into an omega letter shape, Ω, whose trajectory allows for the splitting of the electrons into differentiated channels. Consequently, information on the chemical composition, bonding and coordination states, electronic bandgap, plasmonic oscillations, excitons, etc., can be extracted. EEL spectra may be divided into two distinguished regions, namely low-loss (energy losses below 50 eV) and core-loss (energy losses avobe 50 eV), according to the measured energy lost, providing different types of information. While high energy losses relate to inner electrons close to the atomic nucleus and thus contain chemical information, low-loss analyses involve outer-shell electrons, linked to opto-electronic and vibrational features. We discuss both, separately.

Excitingly, STEM-EELS allows for hyperspectral imaging, meaning that the recorded datasets, also referred as data-cubes, contain one whole spectrum for every scanned pixel, rendering 2D information of the scanned area (i.e., signal maps). Remarkably, EELS analyses may reach a sub-angstrom spatial resolution, along with a high energy resolution, achieving up to a few millielectronvolts if using monochromated electron probes. On one hand, modern instruments equipped with sophisticated electron guns and aberration correctors focus the scanning electrons into sub-angstrom bright probes. On the other hand, the implementation of monochromators at the microscope column, cutting out deviant wavelengths, improves the energy resolution down to <10 meV [49]. Additionally, EELS tomography is possible by acquiring spectral data as tilt series [19].

#### 3.1.1. Core-Loss EELS

The exact amount of energy lost by the electrons irradiating the sample is characteristic of the atomic species constituting the material, providing an accurate analytical tool. Indeed, EELS capabilities are far beyond compositional analyses, as the energy onset and fine structure of the ionization edges (i.e., the shape of the spectral peak) contain information on the oxidation state, local coordination [50], or bonding configuration [51].

**Figure 5:**
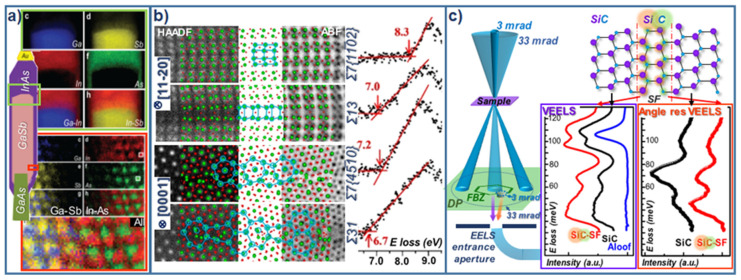
(**a**) InAs/GaSb NW on GaAs stem, combining both axial and radial heterostructure configurations. Ga (blue), In (red), Sb (yellow), and As (green) EELS maps belonging to the light-green (top) and orange (bottom) indicated regions are displayed. Adapted with permission from R.Zamani et al., 2018, Nano Lett., 18, 1557. Copyright 2018 American Chemical Society (ref. [52]). (**b**) Combined use of atomic-resolution imaging (HAADF and ABF) and VEELS, unraveling the reduced band gap (E^bulk^ = 8.8 eV) of different grain boundary configurations for α-Al_2_O_3_. Adapted with permission from J. Wei et al., 2010, Nano Lett., 20, 2530. Copyright 2020 American Chemical Society (ref. [54]). (**c**) VEELS and angle-resolved EELS configurations (right) along with experimental spectra (purple and orange framed, respectively) obtained from SiC (black curves) and from SiC with a stacking fault, SF (red curves). Adapted with permission from X. Yan, 2021, Nature 589, 65. Copyright 2021 (https://www.nature.com/articles/s41586-020-03049-y, 20 February 2020) [55].

EELS measurements allow simultaneous ADF acquisition, and both spectroscopy and imaging may be performed with a sub-angstrom resolution, which is highly important for the study of heterostructures and interfaces. For instance, these sorts of analyses are useful for understanding electronic properties at heterojunctions, whose band alignments are affected by possible diffusion phenomena. The abruptness of interfaces at complex nanostructures is intimately related to the growth mode employed, and the coexistence of abrupt and graded interfaces within individual nanostructures is possible [52]. For instance, the atomic-resolution core-loss EELS analyses shown in Figure 5a evidence atomically sharp radial interfaces in GaSb/InAs NWs, where only one atomic layer shows a contribution from the four constituents (Ga, As, In, and Sb). Accurate chemical analyses are especially desirable when dealing with alloys, since compositional fluctuations and inhomogeneities may also distort the carrier localization driven by band gap variations and related lateral fields, affecting the ultimate performance of devices such as light emitting diodes (LEDs) [53].

In addition to providing chemical identification and quantification, the spectral shape of the ionization edges at the core loss contains information on the bonding and coordination configurations, appealing for the study of atomic arrangements and crucial to fully understanding either intended or undesired doping and related consequences. The techniques applied to the study of single dopants (atomic species) have revealed different bonding configurations of individual atom impurities (i.e., triple- and quadruple-bonded Si dopant within graphene) [56]. As EELS is also suitable for the study of light elements, such as oxygen or nitrogen, it allows for a comparison between their bonding configurations, for instance, as guest atoms in defective graphene, revealing plain differences on their preferred coordination [57].

#### 3.1.2. Low-Loss EELS

The lower energy range of an EEL spectrum reflects opto-electronic features arising from the interaction of the microscope electron probe with the outer electron shells of the atomic species composing the analyzed material. Low-loss EELS (LL-EELS) is therefore also referred to as Valence-EELS (VEELS) and is particularly interesting for the characterization of photonic materials, in order to assess their functional properties. In addition to interband transitions and band gap determinations, LL-EELS proves collective electron oscillations (i.e., plasmon resonances) and as the latest state-of-the-art it has achieved vibrational spectroscopy, resolving spectral features within the IR range.

VEELS band gap measurements of many semiconductors have been reported over the years, including direct and indirect band gaps [58], performed at bulk materials and nanostructures, applied to the study of conventional (elemental and semiconductor compounds –IVs, III-Vs, etc-) and novel semiconductor materilas, as 2D perovskites [59,60,61]. Not only the materials band gaps, but also band aligments can be measured at individual nanostructures if dealing with heterostructure systmes [62]. Other than compositional changes, several factors may influence the electronic structure, such as crystal defects. Interestingly, single grain boundaries have been reported to locally reduced the band gap (see Figure 5b), likely due to the presence of uncoordinated atoms at the defect, addressed by atomic-resolution STEM-VEELS [54]. In fact, many optical parameters can be extracted from VEELS datasets, like the real refractive index n, extinction coefficient k, absorption coefficient α and reflectivity R, from numerically deduced real and imaginary parts of the dielectric function, as reported for GaN nanomembranes [63]. Linked to complementary chemical analyses (by core-loss EELS, for example), VEELS leads the correlation between band gap shifting and compound stoichiometry. In the case of I_2_-II-VI-IV_4_ compounds (namely, Cu_2_ZnSnS_4−x_Se_x_ –CZTSSe-) the varying electrostatic potential induced in non-stoichiometric combinations has been reported to detrimentally shifting the band gap offset [64]. Similarly, oxygen vacancies promote band gap narrowing in ZnO, due to the decresed band gap onset for lower oxygen contents, associated to mechanical deformation [65]. As well as establishing the expected compositional depence of the band gap, these analyses may allow further correlations, such as possible band gap polymorphism depence [62]. Additionally, recent studies have shown the potential of the technique for the analyses of atomic defects in semiconductors, being able to detect and characterize sub-bandgap defect levels, which are key in the later performance of the material [66].

As mentioned earlier, characteristic frequencies of plasmon resonances also lie within the LL-EELS spectral range, driving the extended use of the technique for the study of localized surface plasmon resonances (LSPR) in metal nanostructures, addressing both bright and dark modes, with subnanometer resolution. This fact implies an additional advantage compared to other techniques uncapable of detecting dark modes, such as cathodoluminscence. Although most reported studies deal with noble metal nanostructures with characteristic resonances around the visible range, the analyses may extend over any other system showing collective electron oscillation, from the UV to the IR, providing valuable insights on size and environmental effects, coupling and hybrid modes, etc. The already mentioned technique capabilities allow for exploring different approaches for the study of photonic systems, such as the simultaneous band gap and plasmon map acquisition in ZnO/Zn_1−x_CdxO heterostructures [67]. The methodology can also be successfully applied to the study of other light-matter quasiparticles, such as plasmon-exciton polaritons (plexitons) originating from hybrid systems combining layered semiconductors coupled to metal nanoparticles acting as nanoantennas, probing the plasmon–exciton hybridization, while revealing the plasmon-like charge and field distribution of the plexito [68].

Reaching the IR spectral range, VEELS becomes suitable for the study of phonon related phenomena, only accesible at dedicated aberration-corrected STEM instruments equipped with highly stable monochormators achiving energy resolutions below 10meV [69]. The geometrical optimization of the setup may involve placing the spectrometer off-axis, resulting in DF-EELS configuration, so that localized phonon scattering collection is maximized [70]. Under such conditions, vibrational spectroscopy by means of EELS can be performed even at individual atoms, rendering valuable information on phonon scattering from single insterstial defects. Within this context, red-shifted local phonon resonances associated to single stacking faults have been reported in SiC, [55] by combining high spatial resolution VEELS with angle-resolved VEELS. As acoustic phonon modes reach their highest energy at the edge of Brillouin zone (BZ), the angle resolution can be imporved by filtering the region of interest of the BZ using a cutting-off aperture at the diffration plane (see Figure 5c), providing a setup for angle-resolved VEELS measurements.

Interestingly, the nature of vibrational modes, dependending on the atomic mass rather than on the atomic number, opens the way to isotope resolved studies by means of VEELS, as reported Krivanek et al. [71] Due to the strong impact of isotopic mixing on transport properties, which indeed imposes a further degree of freedom in phonon engineering [72], the future implementation of the approach to isotopic semiconductor materials might allow a better understanding of the underlying phenomena.

### 3.2. Further STEM Spectroscopies

#### 3.2.1. Energy Dispersive X-ray Spectroscopy

Energy dispersive X-ray spectroscopy, EDX, is a well-known analytical technique that can be performed in STEM, allowing for point analyses, line profiles, 2D-maps [73], and even 3D reconstructions [74]. The scanning electron beam induces atomic ionization of the sample constituents by the ejection of inner-shell electrons, which may return to their ground state via X-ray emission. The energy of the emerged X-ray relates to the difference in energy between the two electron shells involved and enables an unequivocal chemical identification. Importantly, the development of X-ray detectors with optimized geometries achieving larger solid collection angles has imposed one striking advancement on these measurements. The X-ray collection efficiency is maximized using multiple detectors (namely silicon drift detectors, SDDs) placed around the sample, resulting in higher sensitivity and faster analyses [75].

Both, EELS (Section 3.1) and EDX are suitable techniques for analytical measurements: while EELS measures the energy lost by the electrons while crossing the sample, EDX gathers the emerging X-rays due to the sample interaction with the electron beam. The detection efficiency of EELS detectors is usually much higher than that of EDX detectors, while the peak-to-background signals for EELS are lower than for EDX. In general terms, EDX is more sensitive to heavy atoms, while EELS is more sensitive to light species. Additionally, the role of the sample thickness must be taken into account, as multiple scattering imposes a thickness threshold (material dependent) for EELS measurements, whilst thicker samples may enhance the X-rays emission.

EDX has been broadly exploited for the analysis of semiconductor materials. The measurements shed light on the composition and abruptness of heterointerfaces, as reported, for instance, for the case of axial and radial metal-semiconductor nanowires, grown from metallic cores created by the thermal exchange reaction of Ge by Al, covered by a thin Si shell [76]. Detailed studies may be performed at atomic resolution [73], rendering information on the spatial distribution of alloyed atoms in ternary semiconductors, such as GaAs_1−x_Bi_x_ [77].

More sophisticated EDX-based methodologies involving further variables, such as the third spatial dimension or the time evolution, are attainable, too. On one hand, EDX tomography retrieves 3D chemical information recording EDX signals for different sample orientations (stepwise tilting of the sample, obtaining one spectrum for every tilt position), as in any electron tomography. The information extracted can be complemented by the simultaneous acquisition of HAADF images, rendering reliable information on the 3D morphology and composition of semiconductor structures [74]. On the other hand, time-resolved compositional analyses are suitable by using in situ approaches (see Section 4).

#### 3.2.2. Cathodoluminiscence

Different material properties may be monitored due to the electron–matter interactions induced while scanning the electron probe over the sample during STEM measurements, such as the emitted photons. An electron beam scanning a semiconductor material may induce electronic excitations creating electron–hole pairs whose recombination leads to photon emission. The measurement of the photon radiative recombination induced by electronic excitations is referred to as cathodoluminiscence, CL, and it is broadly used in scanning electron microscopy (SEM) and is also implemented in STEM. An electron beam scanning a semiconductor material may induce electronic excitations creating electron–hole pairs whose recombination leads to photon emission [78].

CL can be performed under two modes, attending to the acquired spectral range. Collecting photons of any emitted energy leads to panchromatic images, useful for instance to localize active areas. Alternatively, a thin energy window of several electronvolts can be selectively mapped, probing the actual band gap of the analyzed material. This technique finds applications in the study of quantum-confined systems, such as axial [79] and radial [80] heterostructured III-V nanowires. It is worth noting the relevant role of possible interdifussion phenomena or strain, and their correlation with their response, which can be nicely adrressed based on these experiments. The methodology achieves lifetime measurements well below the optical diffraction limit [81]. Bright plasmon oscillation within the VIS-UV range can be also addressed and is suitable, for intance, for the study of single photon emission at point defects in hBN [82]. In addition, the smart combination of CL spectroscopy with other characterization techniques, such as electron microscopy imaging under different modes, EDX, and complementary photoluminescence measurements, enable correlative analysis such as those recently reported for semiconductor hybrid perovskite nanoparticles [83] with enhanced photoeminsision, likely due to quantum confinment effects along with ultralow defects.

Angle-resolved measurements of light emission are also possible, by including a position control pin-hole, as well as the parabolic mirror [84]. Working at aberration corrected instruments at 80 kV, allows forming electron probes with 1 nA beam currents and diameters of about 1 nm, suitable for those studies applied to semiconductor nanostructures.

## 4. In Situ STEM

**Figure 6:**
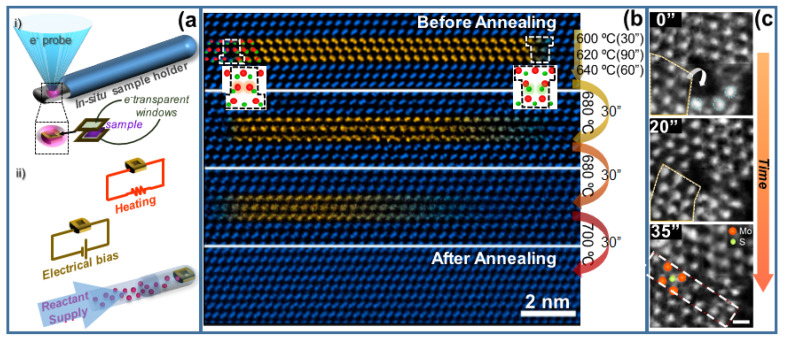
(**a**) Illustration of in situ TEM holders, indicating the location of the sample between two electron-transparent windows (i), and equipped with different kinds of chips and configurations (ii). (**b**) <111> GaAs_0.9_P_0.1_ NW (self-catalyzed, MBE grown on Si) ADF-STEM images of 2 defect configurations (left side and right side, facing 2 Ga and 2 As/P atoms, respectively) during exposure to increasing temperatures. Atomic structure before (top) and after different consecutive heating steps (second-bottom rows) until complete twin removal from the nanowire. Adapted with permission from Nano Lett. 19, 4574. Copyright 2020 American Chemical Society (ref. [90]). (**c**) Reknitting of a lattice hole at MoS_2_ on graphene at 500 °C (elapsed time indicated at the top-left corner): (top) MoS_2_ with a hole. (middle) Hole starts to reknit with diffusion and atomic reconstruction (rototranslational motion of MoS_2_ clusters). (bottom) Reknitted hole, showing twin boundary, as denoted by dashed lines. Mo and S atoms are represented by orange and light-green spheres, respectively. The scale bar is 0.5 nm. Adapted with permission from ACS Omega 6, 21623. Copyright 2021 American Chemical Society [91].

The severe instrumental stability requirements to successfully perform (S)TEM measurements prevent the direct implementation of in situ analyses with conventional instruments, hindering the study of dynamic processes. Fortunately, these limitations can be overcome by specifically designed solutions, which allow for working under different experimental conditions such as varying temperatures, pressures, electric environments, liquid state studies, and so on. The implementation of in situ measurements involves either using environmental TEMs, where the sample chamber works at higher pressures than the microscope column by means of differential pumping, or using specific sample holders that provide the desired environmental conditions for the experiment of interest directly at the sample [85]. In situ holders provide a controlled environment at the sample vicinity, which is isolated from the microscope column by the holder geometry (i.e., the sample is placed between two electron-transparent stacked layers (see Figure 6a) while the microscope vacuum inside the whole column is kept. Based on such a configuration, in situ TEM holders allow for varying not only the temperature, current, or voltage for the sample, but also for supplying gas species to carry out in situ growth experiments. Similarly, liquid-phase measurements require suitable cells to encapsulate the liquid sample between two stacked thin chips with transparent windows at the electron pathway. These TEM cells are usually made of SiN_x_, but different alternatives have shown up during recent years, enabling the production of TEM cells based on 2D-like materials, such as graphene or h-BN for in situ measurements [86,87].

Environmental or in situ TEM measurements have enabled depicting growth dynamics of semiconductor nanowires growing both under vapor–liquid–solid (VLS) [88] and vapor-solid-solid (VSS) growth regimens by tracking the seed and the NW composition during growth. In particular, for the case of Au-seeded GaAs NWs, despite showing comparable growth rates, incubation, and atomic layer completion, they follow opposite trends for the two growth modes (i.e., short incubation and long completion for VLS; long incubation and short completion for VSS) [89]. Likewise, in situ techniques offer the chance for in-depth studies on the creation and/or annihilation of crystal defects, such as the typical crystal twinning/polytypism experienced by III-Vs. Within this context, in situ heating has shown the effectiveness of post-growth annealing for twining reduction in GaAsP NWs since the defects tend to recombine or migrate towards the NW surface. Thus, careful inspection while heating enables one to track the motion of null Burgers vector line defects—twin boundaries (see Figure 6b)—thermodynamically unstable within NWs, with activation energies similar to that of glide dislocations [90]. The formation of twin boundaries has been reported in other semiconductor material systems, such as MoS_2_ grown on suspended graphene by thermolysis, where twin boundaries are created as consequence of the reknitting process to fill holes between neighbor MoS_2_ clusters (see Figure 6c) [91]. There, growth dynamics are addressed by combining in situ STEM imaging with deep learning approaches (see Section 5).

Remarkably interesting is the fact that accessing the materials structure at different experimental conditions brings the chance to explore metastable phases or phase-transition phenomena. As an example, recently published in situ STEM measurements address the structural origin of the metal-to-semiconductor transition observed in PbTe [92].

Annealing processes can also be investigated dealing with heterostructured systems subject to solid-state reactions, such as those reported, for instance, for different Ge-based combinations. In situ experiments have shown thermally induced diffusion phenomena driving by the formation of Ge axial discs thinner than 10 nm within Al NWs, which are thermally induced In the case of the Ge-Cu material system, the annealing process via Joule heating (Ge NWs with Cu contacts) causes both germanium and copper diffusion in opposite directions, resulting in intermetallic phases (“germanidation”) [93].

## 5. Machine Learning and STEM

The implementation of machine learning algorithms has enormous potential regarding the abovementioned techniques, resulting in an invaluable complement to experimental methodologies. Currently, different methods achieve successful structural phase identification, either based on supervised or unsupervised learning approaches [85], which are particularly useful for identifying structural defects. Weakly supervised methods based on the analysis of atomic-resolution STEM images via deep neuronal networks can not only identify a variety of initially unknown defects but also explain complex atomic transformations, such as switching between 3- and 4-fold-coordinated silicon dopants in graphene, as a function of time [94].

Machine learning algorithms also find applications facing spectral data, which is useful, for example, to distinguish the signal sub-space from the noise by dimensionality reduction of EDX data, which in turn improves the identification of light elements (i.e., N) [95], thus offering a promising alernative for the study of semiconductor materials at the nanoscale, such as dilute nitrides.

Combining in situ STEM with a deep-learning framework enables the study of the real-time structural transition from MoS_x_ clusters to a triangular MoS_2_ clusters (see Figure 6c), unraveling the formations of associated structural defects (i.e., mirror twin boundaries) [91]. The method reported in this case relies on the reconstruction of the wave object function from HAADF imaging while heating.

Developing suitable processing strategies becomes particularly beneficial for handling 4D-STEM datasets. A manifold learning machine (i.e., well-established machine learning non-linear techniques) enables relating the extracted experimental patterns to individual atom sites and sublattices, successfully achieving single-dopant recognition, providing an efficient computational tool for image interpretation [96]. Moreover, machine learning approaches may assist the optimization of experimental procedures, for instance, with regards to low-dose imaging by compressed sensing, which allows for minimizing acquisition doses and times [97].

## 6. Overview and Outlook

This review evidences the usefulness of STEM-related techniques for the smart engineering of advanced semiconductor systems. The unique capabilities of STEM, in terms of spatial and spectral resolutions, along with the many meaningful signals arising from the electron–matter interactions, result in a broad range of techniques, finding applications in countless methodologies. Nowadays, technological advances and faster computer-processing capabilities continue pushing the development of improved STEM instruments, driving some of the latest breakthroughs. Furthermore, this review highlights the valuable insights attainable by STEM methodologies applied to semiconductor materials, summarized through recent publications. The gathered examples illustrate the wide scope of characterization provided by STEM, allowing the performance of correlative studies strongly benefiting semiconductor materials research.

The provided overview aims to encourage future in-depth semiconductor STEM characterizations, broadening the understanding of their behavior, alongside the smart engineering of better and novel functional systems.

## Figures and Tables

**Table 1:** Summary of relevant STEM techniques, pointing to the type of collected signal (signal), the detection system requirements and optimal setups (detection), provided information (results) and main limitation (limitation).

Technique			Signal	Detection	Results	Limitations
STEM Imaging	ADF	HAADF	Incoherent elastically scattered e^−^	70–200 mrad	Z-contrast imaging (heavy elements)	Blind to light atoms
		LAADF	Partial coherent elastically scattered e^−^	≈25–60 mrad	Defect contrast	Complex interpretation
	BF	ABF	Elastically scattered e^−^ at the BF	11–22 mrad	Atomic detection of light and heavy elements	Complex interpretation
		MABF	Low-angle scattered e^−^	0–9.5 mrad		Complex interpretation
4D-STEM	Virtual imaging Ptychography		2D-ronchygram arrays	Segmented (quadrant/pixelated) detector	Phase retrieval	Complex data treatmentSample thickness. *
	DPC etc.				Electric/magnetic maps; etc.	
STEM Spectros-copies	EDX		X-rays	Windowless broad solid angle X-ray detector	Chemical analyses	Accurate quantifications Light elements analysis
	CL		Photons	Parabolic Mirror + Photon Spectrophotometer	Radiative recombination (light emission)	Nanometer spatial resolution Blind to dark resonant modes
	EELS	Core-Loss	Inelastically scattered e-(inner shells)	Energy Filter (Dual)	Chemical fingerprint	Complex data treatmentSample thickness
		Low-Loss	Inelastically scattered e-(outer shells)		Opto-electronic and photonic properties	High stability requirements Complex data treatment

* Particularly for some techniques, such as DPC.
